# Educating Engineering Students to Address Bias and Discrimination Within Their Project Teams

**DOI:** 10.1007/s11948-022-00426-w

**Published:** 2023-02-07

**Authors:** Siara Isaac, Nihat Kotluk, Roland Tormey

**Affiliations:** École Polytechnique Fédérale de Lausanne (EPFL), Centre for Learning Sciences/Teaching Support Centre, 1015 Lausanne, Switzerland

**Keywords:** Bias, Discrimination, Ethics, Teamwork training, Emotion, Project-based learning, Microaggressions

## Abstract

What training should engineering students receive to enable them to contribute to reducing bias, discrimination and the persistent lack of diversity in engineering? Collaboration is central to professional engineering work and, consequently, teamwork and group projects are increasingly present in engineering curricula. However, the influence of unconscious bias on interactions within teams can negatively affect women and underrepresented groups and is now recognised as an important engineering ethics issue. This paper describes a workshop designed to enable engineering students to work equitably in diverse teams. Key features of the workshop include (1) the emotionally safe, empowering and warm environment created, (2) the creation of opportunities for students to discuss and apply the issues raised to their own engineering projects, and (3) the opportunities to practice the use of both proactive and reactive strategies to address bias and discrimination in teams. The evaluation of the workshop suggests that engineering students regarded both onsite and online formats as providing useful skills that they intended to apply in practice. Follow-up evaluations suggest that the workshop leads to behavioural change, especially the use of proactive teamwork strategies intended to reduce the impact of unconscious bias.

**Supplementary Information:**

The online version contains supplementary material available at 10.1007/s11948-022-00426-w.

## Introduction

Addressing persistent issues of equity and discrimination in engineering can appear to be an intractably big issue. Like any wicked problem, no single solution will solve it. However, we believe that one valuable piece is present in our initiative to equip students with skills to reduce bias in interactions and address potential microaggressions that occur during teamwork. This paper describes a short, scalable workshop for engineering student teams focusing on providing them with (1) practical strategies for working together more equitably and (2) opportunities to apply and reflect on an initial experience using the tools. While firmly contextualised in engineering, our approach attends to both the emotional climate of the workshop and to the interactions between bias and emotions, therefore how emotions influence decision-making and collaboration in teams. Collaboration is central to professional engineering work and, consequently, teamwork and group projects are increasingly present in engineering curricula in order to offer students opportunities to develop the necessary skills (e.g., Borrego et al., 2013; Crawley et al., 2014). But there is growing recognition that unconscious bias affects interactions between people in teams and groups, which can have a negative impact on the emotional well-being of colleagues and fellow students (e.g., Swim et al., 2003) and on their learning (Aeby et al., 2019). In engineering education settings, this in turn contributes to the *chilly climate* which is an important factor in the under-representation of some genders, sexual orientations and ethnic groups in the engineering profession and in engineering education (Barnard et al., 2012; Lichtenstein et al., 2014; Meadows et al., 2015; True-Funk et al., 2021).

The patterns of actions and decisions arising from these unconscious biases are increasingly recognised as an important ethical issue for engineers. The IEEE code of ethics (2020), for example, says members undertake “to treat all persons fairly and with respect, and to not engage in discrimination based on characteristics such as race, religion, gender, disability, age, national origin, sexual orientation, gender identity, or gender expression.” Similar statements appear in other recent codes, including the Association of Computing Machinery (ACM, 2018) and the US National Society of Professional Engineers (NSPE, 2019). As part of their ethical framework, engineering programs must respond by training students to work together equitably during their studies and professional life.

The next section of this paper provides an overview of the current thinking on unconscious bias as an ethical issue in engineering education and educational strategies to address this issue. The following section is the heart of the paper; it provides a rich description of the workshop with the intention that others may find the activities and the approach underpinning them relevant to inform the training students receive in their institutions. The final section describes the evaluation of the workshop and discusses the implications of the participant feedback. It shows that the workshops appear to have a positive impact, that is sustained in online as well as in face-to-face formats, and that proactive strategies may be particularly important to include in such ethics training.

## Developing Equitable Teamwork Skills

Although engineering is a technical profession, the collaborative, applied nature of engineering work demands that engineers are able to work in diverse, multidisciplinary teams (Docherty et al., 2020; Miranda et al., 2019). Indeed, these skills are expressly required by many engineering accreditation agencies (ABET, 2013; ENAEE, 2021). Educational researchers, faculty, and employers concur that team projects in engineering are a key way to develop these skills in undergraduate students (Paretti et al., 2014; Borrego et al., 2013; Isaac & Tormey, 2015; Crawley et al., 2014).

While team projects offer students the opportunity to interact in groups, issues can arise. For example, Kelly Cross and Marie Paretti (2015) found that personal interactions directly influenced how black students experienced their student teams in engineering and Prisca Aeby et al. (2019) found that gender influenced expectations and task assignments in student engineering teams. While the difficulties underrepresented groups encounter in engineering student teams have been identified (Cech & Rothwell, 2018; Dee & Gershenson, 2017), there is less work investigating how conflict, bias, and inequality are managed by student teams (Aeby et al., 2019; Isaac & Tormey, 2015; Miranda et al., 2019; Strachan et al., 2018; Wolfe et al., 2016).

One factor which has been identified as particularly important in a wide range of social settings is *implicit* or *unconscious* bias. As the name implies, unconscious bias refers to introspectively unidentified (i.e. unconscious) stereotypes that affect the qualities that we attribute to or expect (i.e. bias) in particular social categories (Greenwald & Banaji, 1995). Unconscious biases frequently conflict with the beliefs that people describe as their own (Lai et al., 2013), exerting their influence in quick assessments that can precede more intentional or analytical thought (Strachan et al., 2018). The influence of unconscious biases is therefore often subtle, working at the level of pre-reflective micro-decisions or behaviours which set up our subsequent reactions, attitudes, or decision-making (Bock & Welle, 2014; Sue et al., 2009). Even when people are consciously trying to be fair or objective, unconscious biases may affect their decision-making, particularly in situations of stress or urgency (such as those under which engineering student teams often work). While typically subtle, the cumulative effect of unconscious biases can reinforce and perpetuate damaging stereotypes (Atewologun, Cornish & Tresh, 2018; Dee & Gershenson, 2017; Jackson et al., 2014; Kahneman, 2011; Strachan et al., 2018).

Stereotypes and prejudices have been shown to negatively affect the experience of groups underrepresented in engineering education programs (Fouad et al., 2017; Jackson et al., 2014). The negative impacts include exclusion, loneliness, health problems, and discrimination in team projects (Cech & Rothwell, 2018; Cross & Paretti, 2015; Dee & Gershenson, 2017). For example, encounters with hegemonic masculinity before and during engineering programs negatively affected the experiences of both openly gay and female engineering students (Hughes, 2017). Experiencing *chilly climates* is one of the reasons why members of underrepresented groups leave engineering education (Chen & Thomas, 2009; Dennehy & Dasgupta, 2017; Farrell & Minerick, 2018; Geisinger & Raman, 2013). Negative effects of student–student bias in engineering programmes can lead to exclusion of underrepresented groups from high-profile team roles (DeCosta et al., 2020), increased need for underrepresented groups to prove their intellectual worth to their team (McGee & Martin, 2011; Smith & Gayles, 2018), lower social inclusion among their peers (Marra et al., 2009), and being targeted by micro-aggressions from peers (Li et al., 2017; Yang & Carroll, 2018). The cumulative result is that students subject to bias experience higher rates of academic failure (Farrell & Minerick, 2018). These negative experiences of underrepresented groups contribute to the persistent lack of diversity in engineering programs (True-Funk et al., 2021).

Historically, these issues of bias and discrimination have been seen as outside of the domain of engineering ethics (Rottmann & Reeve, 2020); indeed, Barry and Herkert (2014, p. 686) describe them as having been “not considered in traditional engineering ethics education.” Recently, however, there has been a concerted focus on these issues in engineering ethics, as seen in their inclusion in revised codes of ethics from ACM (2018), IEEE (2020), and NSPE (2019), as well as in academic literature (e.g. Barabino et al., 2020; Rottmann & Reeve, 2020). Nevertheless, there are challenges to integrating such issues into ethics education. Traditional approaches to ethics education tend to emphasise a rationalist approach to reflection on ethical issues (see Tormey, 2020) coherent with the development of students’ *ethical sensitivity*, the ability to recognise that an ethical concern exists in the first place, and *ethical judgement*, the ability to apply principles in a reasoning process and come to judgement (Narváez & Rest, 1995). These are certainly important skills; however, there is emergent recognition of the importance of rapid, emotional and unconscious appraisals in engineering ethics (Gelfand, 2016; Hess et al., 2019; Roeser, 2007, 2010, 2012). Unconscious bias poses a particular challenge precisely because its impact occurs in rapid, pre-reflective, and unconscious micro-decisions that can precede or subtly flavour the construction of the logical reasoning path targeted by traditional ethics teaching. Addressing unconscious bias requires emotional work alongside cognitive work to advance the learning aims of *ethical motivation*, the internal drive to prioritise behaviours underpinned by ethical values over other values such as career or institutional loyalties, and *ethical character* (or *ethical agency*), the ability to manage one’s own psychological resources and to work within social and organisational settings to follow through with ethical behaviours (Bebeau, 2002; Narváez & Rest, 1995). Whilst student trainings targeting students’ ethical sensitivity and reasoning have been previously reported, the strategies and activities designed to develop these latter two skills of *ethical motivation* and *ethical agency* constitute the major contribution of our approach.

Given the ethical implications of bias and discrimination, unconscious bias training (UBT) should be included in engineering students’ ethics education. UBT typically addresses biases related to aspects of people’s identity, such as gender, sexual orientation or nationality. Training may also present strategies to reduce the influence of such biases on their interactions with others (i.e., *ethical agency*) (Atewologun et al., 2018; Girod et al., 2016; Nelson, 2017). Most UBT are in workshop form, either face-to-face or online. Self-testing for implicit biases is frequently included, often by asking participants to complete a computer-based response-time test which is designed to assess unconscious associations between social categories and stereotypical attributes (such as the implicit bias tests from Harvard’s Project Implicit—http://projectimplicit.org/index.html) (Greenwald et al., 1998).

UBT has been shown to increase participants’ awareness and knowledge of diversity issues (Atewologun et al., 2018; Bezrukova et al., 2016; Herbert & Will, 2021; Jackson et al., 2014), which is integral to developing *ethical sensitivity* around these issues. However, like much ethics education, the follow through into ethical action remains questionable. Some studies have found that some formats of UBT have a limited impact on biased behaviour (FitzGerald et al., 2019; Forscher et al., 2019; Green et al., 2019). There is not yet a consensus on educational approaches that move beyond sensitivity to impact on behavioral change (Atewologun et al., 2018; Foley & Williamson, 2018; Green et al., 2019).

Many of the studies cited in the previous paragraphs refer to UBT in a range of different academic and employment settings. There have also been some attempts to address these issues specifically in engineering education. Sheridan et al. (2021) studied the impact of a UBT workshop for faculty 6–12 months after the workshop and found that it had an impact on sensitivity to bias issues and discussion of bias issues, but did not impact on self-reports of behaviour. Similarly, Chromik et al. (2020) found that training with students showed students increased awareness of the potential impact of implicit bias. The impact of self-reported behaviour was, however, more mixed with the percentage of students who felt the training impacted their teamwork varying between 14 and 63% across the four courses.

Encouragingly, some studies identified an impact beyond ethical sensitivity. O’Leary et al. (2020) reported that a faculty workshop led to an impact on both sensitivity and on motivation to address social equality issues in teaching. Rooney et al. (2020) found that online training led to enhanced faculty self-confidence in relation to managing bias in their classes, which suggests increased ethical agency as well as sensitivity. Issues identified as relevant to the success of both were the facilitation of meaningful dialogue that allowed participants to construct understanding linked to their own experiences, and a focus on concrete strategies for action. Similarly, in work with engineering students, DeCosta et al. (2020) found that providing concrete strategies for teamwork led to self-reported higher team focus on interpersonal areas such as offering and receiving feedback, discussing ideas, making decisions, and justifying evidence-based design decisions when compared with a control group.

Overall, prior work in this area highlights that unconscious bias is a significant ethical and social justice issue in engineering education. It also highlights the need to move beyond purely cognitive and reasoning-based models for ethics education to address this issue effectively. There is reasonable evidence that forms of UBT can be effective in addressing participants’ awareness of and sensitivity to bias issues. There is more limited evidence that it is possible to increase ethical motivation (to address bias) and ethical agency. While there is very limited data on this at present, the evidence that exists suggests that supporting the development of appropriate behaviours and strategies, aligned with opportunities for meaningful dialogue and constructivist learning, may well be important in effective unconscious bias education. The approach of our workshop is informed by this research.

## Taking Action on Unconscious Bias in Engineering Student Teamwork

### Positionality of the Authors

Secules et al. (2021) have argued that when addressing issues of equity in engineering education research, being clear about authors’ positionality is important for reflecting on and dislocating privilege. We therefore begin this section by locating ourselves as authors. This workshop originated as the personal project of one of the authors (Siara Isaac) who was interested in understanding and addressing discrimination and persistent bias in society. Working as a pedagogical advisor and trainer meant that educational activities were a natural choice both for intervening in the world and for developing her own knowledge and skills. As the workshop developed, she was asked to facilitate similar workshops with students on campus. The philosophy of the educational team in which both Siara and Roland work is to engage in evidence-informed educational practice, which is subject to rigorous evaluation and where the results are shared with the wider community. This philosophy influenced the design of the evaluation of the workshop: Our methodology for assessing the workshop’s impact is an adaptation of a method common among pedagogical advisors and one we have used previously in other projects (Tormey et al., 2019).

Since Siara developed and facilitated the workshops, the first person singular (‘I’) is used in the rest of this section to describe the workshops which she facilitates. Other members of the writing team (Nihat, Roland) contributed to developing the pedagogical approach and analysis of the impact of this workshop. The first person plural (‘we’) is used in the rest of the paper to refer to the writing team as a whole.

### Approach of the Workshop

This section presented the core contributions of the paper and describes the goals and structure of the workshop, paying particular attention to (1) the way in which the emotional climate of the workshop was managed (*ethical sensitivity*), (2) how the effects of unconscious bias were made relevant to engineering students (*ethical motivation*), and (3) how strategies for preventing and addressing bias in student engineering projects were taught (*ethical agency*).

The workshop described here was compulsory for students who wished to participate in one of several flagship interdisciplinary engineering projects in our engineering school. The three core goals for the workshop are to (1) increase awareness of bias in decision-making and interaction for collaborative work in engineering, (2) provide strategies for decision-making and collaborative work that mitigate bias, and (3) providing opportunities for students to practice these skills in situations which approximate those they are likely to encounter in engineering projects. The first goal is clearly linked to *ethical sensitivity*, while the second and third goals support *ethical agency* by equipping students with practical tools and practice using the tools in team settings. The workshop was originally taught in an onsite face-to-face format. However, the COVID-19 pandemic temporarily imposed an online format (along with all other teaching). While this move limited face-to-face interaction, the workshop format remained essentially unchanged due to the use of online tools and virtual breakout rooms. The workshop activities fall into 5 phases, shown in Table 1.

**Table 1:** Outline of the workshop activities

	Phase	Activity	Details
1	Creating a constructive environment for the workshop	Welcome	Foregrounding the *ethical motivation* for the workshop themes and establishing a constructive climate
2	Acknowledging the effects of bias in engineering	Take and reflect on an *Implicit Attitudes Test*	Generating *ethical sensitivity* through building awareness of bias that is largely outside of our conscious awareness https://implicit.harvard.edu/implicit/
3	Motivations and proactive strategies for equitable teamwork in engineering	*Space Ark* activity using decision-making strategies in a team	Developing *ethical agency* through identifying proactive strategies for dealing with bias, and then practicing them in a decision-making activity
4	Micro role-plays on reactive strategies for challenging discriminatory behaviour or comments	*Make It Awkward* activity	Developing *ethical agency* with short, repeated opportunities to increase our repertoire of responses for speaking up when we see or hear discrimination
5	Conclusion	Reflection, wrap up	Developing *ethical agency* by identifying concrete actions to implement in our project teams Evaluation questionnaire

#### Creating a Constructive Environment for the Workshop (Phase 1)

The goal of this first phase is for students to connect with their pre-existing values about equity and to become more aware of the importance of working to increase equity on campus. Confronting our own biases can be unpleasant. Hence establishing a positive emotional environment for the workshop is essential. It can be helpful to think of educational relationships in higher education in terms of 3 socio-emotional dimensions; *affiliation, attachment* and *assertion* (Tormey, 2021; Tormey & Isaac, 2021; Tormey, Le Duc & Isaac, 2022).

*Affiliation* is the sense of liking and warmth communicated in the workshop. For on-campus workshops, this was fostered through setting up the small islands of tables, providing cookies to students, and through a conscious effort to communicate an informal and warm presence throughout.

*Attachment* refers to feelings of security and trust. Students feel safer with the instructor when they are confident that they will not be embarrassed or ridiculed and when the instructor respects the conditions set out for the relationship. One central technique used was to get students to work in smaller, stable groups that created opportunities for students to get to know a couple of other people and to share their ideas within these smaller groups. I avoided asking participants to share their own experiences since this potentially exposes aspects of their identity or experiences that could be, or feel, unsafe for students. While participants clearly do sometimes spontaneously share their stories, this is at their own initiative, peripheral to the activities, and usually within their small group. Another strategy for supporting psychological safety is the use of an electronic classroom response system (CRS) with multiple-choice questions to allow students to reply anonymously to questions during whole-class discussions. This method was consistently used for anything potentially embarrassing, such as how students felt about their implicit bias score (options included proud, annoyed, curious) or checking understanding of concept definitions during the workshop.

*Assertion* refers to the sense of power or status that students attribute to themselves and to the teacher. It was important that students see me as having the authority to be listened to and also see themselves as being empowered in the process. I find myself attempting to establish my authority/credibility through my STEM background (MSc), as an educational researcher (Ph.D.), and 9 years of training professors in the institution yet as a learner in terms of the workshop skills. For example, I share examples where I have unintentionally committed biased acts or missed opportunities to speak up to discriminatory comments. My intention with this balance, between presenting myself as a competent professional yet allowing that equity draws on different skills, is to support students’ *ethical agency*. I want to demonstrate to students that people can make meaningful changes to their own behaviour and to the institutional climate. A key point that I reiterate is to frame the difficulties or discomfort that we encounter from workshop activities not as a failure but as part of the learning and, therefore, integral to developing new skills.

While establishing a constructive relational environment is key in the first phase of the workshop, ensuring it remains so is a central concern throughout the workshop. As we progress through the activities outlined below, I try to respond to participants’ various levels of *ethical sensitivity* and *agency* to challenge them, yet not push anyone too far. I find this workshop quite tiring to teach.

#### Acknowledging the Effects of Bias in Engineering (Phase 2)

Phase 2 aims to increase students’ awareness of the measurable impact of bias and how it can exert an influence (even on our technical and analytic thinking) despite our intentions. Seeing our own bias is, of course, incredibly difficult. Having students complete an online *implicit bias test* (as discussed above) allows them to get feedback that helps them “see” their own bias. I follow up with examples from engineering where unfounded assumptions have led to poor design outcomes. I then present studies (Goldin & Rouse, 2000; Hoffman et al., 2016; Milkman et al., 2012) to illustrate how implementing some of the proactive strategies, summarised in Table 2, decreased inequity.

**Table 2:** Proactive strategies to mitigate the impact of bias on engineering teamwork and decision-making

Awareness of human propensity for bias
Intention to improve
Using explicit criteria for decision making
Hiding irrelevant information
Reducing arousal (stress, fear, etc.)
Collecting data to monitor outcomes, results or processes

#### Proactive Strategies for Equitable Teamwork—Space Ark (Phase 3)

Phase 3 aims to develop students’ toolbox of proactive strategies for managing bias and therefore improving the quality of their decision-making and team interactions in engineering projects. Students first deepen their understanding of the value of diversity in teams and proactive strategies to address bias using an interactive peer teaching method called jigsaw (Aronson & Thibodeau, 2006). The support material for the jigsaw activity is one-page summaries of either research into the value of team diversity for decision-making or specific strategies for equitable teamwork. Students then complete a group activity (called *Space Ark*) that provides them with an opportunity to apply the strategies. This task was chosen because it requires students to find solutions in the face of constraints and to make collaborative decisions, both of which are required for their engineering team projects.

*Space Ark* is a decision-making activity where student groups must decide, under time pressure, who from a list of designated possible survivors to send on the last spaceship leaving Earth before its destruction. The list contains basic demographic information for each person, for example, “31-year-old male engineer with a physical disability (nature of the disability is not provided).” One student acts as an observer in each group, using a rubric to record the group’s discussion (Does the team use any decision-making tools?), interaction patterns between students (Who monopolises talk time? Whose ideas are written down?) and assumptions (What stereotypic things are said about the people on the list?). The post-activity debriefing in the small group, drawing on the observer’s record, helps students to identify some assumptions or poor decision-making that occurred during the activity and to reflect on where proactive strategies could have been used to improve the quality of their decision-making and interactions. I explicitly introduce this decision-making activity as an opportunity for students to put into practice some of the strategies; however, students are generally caught up in the intellectual challenge of the artificial dilemma posed and often do not apply many of the strategies. Students’ surprise at how quickly they lose sight of their intentions to apply proactive strategies creates excellent conditions for reflection and learning. This means that the activity, in addition to providing information about how and when to use specific decision-making or collaboration strategies, also helps students see the difficulties of implementation. This phase of the workshop targets students’ *ethical agency*, giving them direct and immediate feedback on their collaborative decision-making strategies in a low-stakes environment accompanied by an opportunity to consider when and how to apply the strategies in their own engineering projects.

#### Reactive Strategies for Speaking Up to Discriminatory Behaviour or Comments—Make it Awkward (Phase 4)

 The penultimate phase aims to equip students with strategies to react effectively by *Making it Awkward* if people engage in discriminatory speech or acts. The main activity in this section is a three-person role-play activity targeting *ethical agency*. Students are presented with a set of eight possible responses that bystanders can use to challenge problematic behaviour in a non-confrontational way. I give these to students on pocket-sized cards they can keep with them. For example, one strategy is to intentionally misinterpret a biased statement as if it has an implicitly positive intention. One phrasing of this could be, “*Yeah, there are a lot of immigrants here. I really like how diverse it is here. You get people from all over, it’s great*”. Students have an opportunity to start translating the strategies into their own words, first in writing and then speaking aloud. Finally, students undertake role plays in which they practice using the eight strategies to respond to examples of discriminatory speech they may hear.

The role-play allows participants to practice speaking up, and getting feedback from each other, by taking turns playing the role of the instigator (who makes the problematic comment), the responder (who responds) and the observer. Different strategies feel more accessible to some people or in some situations, so I suggest that students use this opportunity as a “sand box” to experiment and try out responses that they do not usually use. This 3-person activity is informed by work in counseling and psychotherapy education (Crowe, 2014; Smith, 2016). Each round of the role-play typically takes 2–3 min (see Table 3), and participants often need to be encouraged to keep their discussion short and to dive back into another (uncomfortable) round of practice.

**Table 3:** Reactive strategies role-play–the 4 steps in each round

Step 1: The *instigator* reads a (mildly) discriminatory comment
Step 2: The *responder* calls them out, using consecutive opportunities to try out different strategies
Step 3: The *instigator* reacts constructively to being called out, using a three-part script to take responsibility for their comment
Step 4: The *observer* watches the interaction and provides feedback to both the *responder* and *instigator*

The biased comments are mostly drawn from students’ accounts of their experiences in engineering student projects, formulated to touch a wide variety of discriminatory opinions (transphobia, xenophobia, ableism, in addition to sexism and racism) yet remain mild enough to avoid being strongly triggering. A key motivation for providing these prompts is to avoid offensive adlibbing, or asking participants to re-live difficult moments in their life by sharing their own experiences. The practice afforded by the role-play seeks to develop students’ *ethical agency*, providing them with practical strategies and practice that can help them be more able to respond more often when they hear or see discrimination.

#### Wrap Up (Phase 5)

The wrap-up aims to develop *ethical agency* by creating a link between the workshop and participants’ future actions. I ask participants to review their experiences in the workshop and to identify which strategies they would like to try to use in practice in their engineering projects. Part of this reflection process is a workshop evaluation survey. In the following section, we evaluate our approach using participants’ feedback collected with this survey and their self-reported behavioural changes several months later.

## Assessing the Impact of the Training

While the primary contributions of this paper are found in the preceding descriptions of the workshop activities and approach, we sought to investigate if our pedagogical choices were indeed effective in achieving our stated learning objectives around *ethical motivation* and *ethical agency*. This final section briefly presents some participant feedback and discusses the implications for training engineering student project teams. Interested readers can find additional feedback data in the Supplemental Information.

### Participants

In total, 382 engineering students attended the workshop between March 2020 and November 2021. Workshop registration data showed both Bachelors and Masters students participated and that they came from a range of study programmes, including chemistry & chemical engineering, civil engineering, electrical engineering, computer science, life science engineering, mechanical engineering, and robotics.

For onsite workshops, evaluation surveys were distributed on paper at the end of the workshop. The survey asked for no identifying information, but participants were asked to self-address an envelope so that they could be sent a follow-up questionnaire some months after the initial workshop. An anonymous code allowed the data from the two surveys to be paired. For online workshops, an anonymous online questionnaire was sent to participants but logistical constraints prevented the administration of follow-up questionnaires. The number of participants, completed questionnaires, and workshop formats are presented in Table 4.

**Table 4:** The number of participants, completed questionnaires, and workshop formats

Workshop period	Format	Attendees	In-workshop questionnaire responses	Follow up questionnaire received
March 2020	Campus	68	61	13
October 2020	Campus	104	104	29
March 2021	Online	106	44	–
October 2021	Campus	104	104	17
Total		382	313	59

### Measures

It is common practice to assess participants’ reactions at the end of training workshops (Alliger et al., 1997). It is less common to go beyond reactions to assess whether participants’ see the workshop material as something they want to implement themselves. When participants perceive that the workshop enables them to achieve their own goals, they make positive *utility judgements* about the material. For readers familiar with Kirkpatrick and Kirkpatrick’s model (see Alliger et al. (1997) for an overview), participants’ reactions after a training are considered level 1 evaluations, whereas utility judgements approach level 3 evaluations. In our context, participants’ utility judgements describe the degree to which they found the activity useful to achieve their current goals (as engineering students) or future goals (as engineers). While the ultimate goal of our workshop is behavioural change, participants’ utility judgements are useful since utility judgements have a stronger correlation with participants’ transfer into their practice than do direct tests of participants’ learning at the conclusion of a training session (Alliger et al., 1997) (for a discussion on the costs and benefits of different methods of workshop assessment see Tormey et al., 2019, pp. 388–389). The exit questionnaires that students completed immediately after the workshop, in addition to several reaction-type questions, included utility judgement items about students’ intentions to use the skills in practice. Follow-up questionnaires that students in onsite workshops mailed back to us several months after the workshop asked them to self-report on their behaviour changes.

The response rate for the exit questionnaire for the campus workshops (98%) is excellent, while the response rate for the online exit questionnaire for the online workshop (42%, 44 out of 106) is within the normal range for online questionnaires (Saleh & Bista, 2017). Responses were collected on a 4-point Likert scale with “no opinion” placed separately on each line.

### Students’ Utility Judgements on the Campus and Online Editions of the Workshop

**Chart 1:**
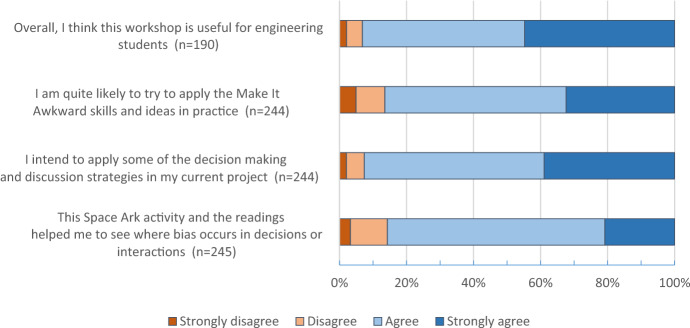
**Onsite workshop students’ utility judgements and intention to use (per exit survey)**. Note: Total n = 269 for 272 on campus participants, missing or no opinion responses are excluded in this chart. The overall utility question was added in October 2020 and so has a total possible n = 208. Words in square brackets are included to facilitate the reader’s comprehension and were not part of the original items presented to students

Participants’ reactions to the workshop activities were very positive (Supplemental Information, Charts 4 + 5) and, more importantly, their utility judgements indicated they intended to use the strategies from the workshops. Although the content and format of the workshop are radically different from the technical courses that students normally attend, and despite the fact that attendance was compulsory for the students, 93% of respondents who attended on campus agreed that the workshop is useful for engineering students (Chart 1). A similar percentage agreed that they planned to try to apply the strategies for managing bias in team discussions and decision-making. A strong majority of participants responded (86%) that phase 3 of the workshop (*Space Ark*) enabled them to see in concrete terms where bias occurs during their own teamwork and also reported an intention to apply reactive strategies in their project (*Make it Awkward*). We attribute this strong result to the powerful emotional and cognitive dissonance caused by having data about their own collaborative practices and, to a lesser extent, data from published studies, that challenged their perception of their collaboration and decision-making practices.

**Chart 2:**
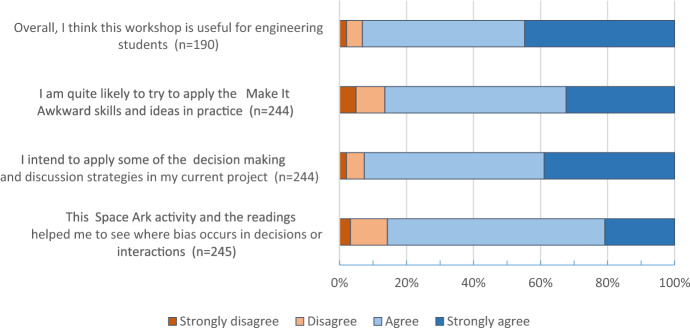
**Online workshop exit-survey utility judgements and intention to use.** Note: Total n = 44, missing responses are excluded in this chart. Words in square brackets are included to facilitate the reader’s comprehension and were not part of the original items presented to students

Perhaps surprisingly, the data for the online workshop (Chart 2) showed a pattern of positive utility judgements similar to that of the onsite workshop (as well as positive reactions; Supplemental Information, Chart 5). Over 95% of online respondents agreed that the workshop is useful for engineering students, all of those who answered indicated that they were quite likely to try reactive strategies (*Make it Awkward*) and over 90% agreed that the *Space Ark* enabled them to see concretely where bias occurs (albeit with about one-quarter who did not answer this question). Even with the context of the typically low response rate (42%), we found it surprising that a workshop that is so interactive and relational seemed to work as well—if not better—online as compared to onsite. We therefore explored this apparent difference and found (Supplementary Information, Table 5) that the utility judgements and intention to use the strategies were statistically significantly higher for the online workshop at the 0.05 level. One possible explanation is the non-response bias of less enthusiastic online participants; however, this effect has not been shown to alter the mean values for course evaluations (Stowell et al., 2012). Our hypothesis is that students who took the workshop online during the covid-19 pandemic were also working remotely with other students on their engineering projects; in this unfamiliar online environment, students found it difficult to apply their usual face-to-face collaboration and decision-making strategies. Students were not accustomed to online teamwork and therefore judged the usefulness of the practical, equitable strategies provided by the workshop even more positively than did campus students.

### Students’ Behavioural Change After the Workshop

As noted above, we followed up with a mail survey some months after the onsite workshops to see if students self-reported any behavioural changes. Responses were collected on a 5-point Likert scale. About one-quarter of respondents returned these follow-up questionnaires, a value significantly higher than the 10% typically observed for personalised postal surveys (Sinclair et al., 2012). Despite higher than expected response rate for a mail survey, not having responses from all participants is a potential source of bias: it is possible that the most enthusiastic participants were those most likely to return the follow-up questionnaires (or the most unhappy) which would give an overly positive (or negative) view of the experience. Thus, we assessed the similarity between the exit questionnaire responses of the 59 participants who returned follow-up questionnaires and the responses of participants whose follow-up questionnaires were not returned. Our statistical analysis (Supplemental Information, Table 6) showed the two groups to be very similar, which allowed us to take the returned questionnaires as broadly representative of the participants more generally.

**Chart 3:**
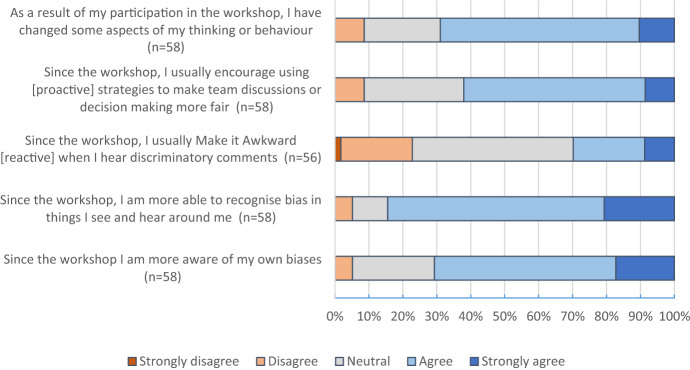
**Students’ reports of behavioural changes after several months.** Note: Total n = 59, missing responses are excluded in this chart. Words in square brackets are included to facilitate the reader’s comprehension and were not part of the original items presented to students

As shown in chart 3, 71% of the follow-up respondents stated that the workshop increased their awareness of their own biases (e*thical sensitivity*), 69% said that they changed some aspects of their thinking or behaviour as a result of the workshop, and 84% reported being more able to recognize prejudiced things they see and hear around them (e*thical sensitivity*). While the questionnaires completed at the end of the workshop indicated that students intended to try reactive strategies (such as those from the *Make It Awkward* activity), students’ follow-up questionnaire responses indicate that proactive strategies (such as those from the *Space Ark* activity) were easier for them to put into practice: 62% reported suggesting that their team use proactive strategies to make team discussions or decision making fairer. Only 30% of the follow-up respondents stated they applied reactive *Make it Awkward* strategies when they heard discriminatory comments (47% gave a neutral response to this item). A rosy interpretation of this lower percentage of engagement with reactive strategies could be attributed to a lack of opportunities; that is, while all students had opportunities to work in groups, not all heard discriminatory comments that required a response. Nonetheless, participants’ follow-up responses indicate that some students (23%) do not usually react to such comments. This may suggest that, even though students were enthusiastic about reactive approaches at the end of the workshop, they found proactive strategies easier in practice.

While generic bias training shares many features with our workshop, the highly applied and contextualised nature of our activities was carefully developed to support participants with the key difficulty of transferring the strategies into their own project work. Based on their responses, our participants increased their *ethical sensitivity* and *ethical agency* for managing bias in decision-making and collaboration. Surprisingly, the online workshops seemed to be as successful as the onsite workshops. Follow-up data suggests that many students have applied the strategies in practice. In particular, proactive strategies appear to have been used most often by students.

## Conclusion

Issues of discrimination and bias based on race, religion, gender, disability, age, national origin, sexual orientation, gender identity, or gender expression are pernicious and multifaceted, resulting in important ethical issues for professional engineers despite not being “considered in traditional engineering ethics education” (Barry & Herkert, 2014, p. 686). As discrimination often seems to arise from implicit or unconscious bias, engineering ethics education should involve activities that develop the cognitive and emotional skills underpinning the *ethical motivation* and *ethical agency* necessary to follow through on ethical intentions. This paper presents workshop activities designed for engineering students working on team projects, where they can directly apply the strategies.

Our approach prepares engineering students to be attentive to both the interactions between bias and emotions, and to how their emotions influence their decision-making and collaboration in teams. In the workshop, we create opportunities for students to experience how their emotion and intuition influence their decision-making, to reflect on and discuss what that means for their design thinking and their interactions with others, and to practice using both proactive and reactive strategies to address discrimination and bias in student project teams. Results show that the workshop seems to achieve its goals: students are more aware of their biases and its impact on their interactions, they learn strategies to address these biases, and they regard the strategies as being helpful to them as engineering students. Evaluation results suggest that the workshops had a particularly positive impact for those engaged in online student project teams. The results also suggest that students may have found it easier to enact proactive strategies to minimise the risk of biased outcomes rather than relying on reactive strategies to address discriminatory behaviour after it occurred. This assumes that students did encounter opportunities when they could have applied the reactive strategies; as noted above, our questionnaire format means we do not have data to test this. Future studies looking at the impact of similar programmes should therefore consider asking students whether they experienced situations in which reactive strategies may have been applied, as well as asking them about their use of such strategies.

In an engineering education context where discriminatory speech and micro-aggressions contribute to the *chilly climate* that perpetuates the under-representation of women and other groups in the engineering profession and in engineering education (Barnard et al., 2012; Lichtenstein et al., 2014; Meadows et al., 2015; True-Funk et al., 2021), equipping engineering students with strategies to reduce the impact of such biases, and giving them the opportunity to practice them, needs to become integral to the practice of engineering ethics educators. Graduating engineers that are well equipped with strategies to manage bias and discrimination in their collaborative work and decision-making would not only have a positive impact on the diversity of this group itself, but also on the hiring, culture and decision-making of their future employers.

## Supplementary Information

Below is the link to the

Supplementary file1 (DOCX 33 kb)
