# Supplementary material for: Educating Engineering Students to Address Bias and Discrimination Within Their Project Teams
Source: Sci Eng Ethics. 2023 Feb 7;29(1):6. doi: 10.1007/s11948-022-00426-w (PMC9903283; doi:10.1007/s11948-022-00426-w)
Supplement: Supplementary file 1 — Supplementary file1 (DOCX 33 kb) [file 11948_2022_426_MOESM1_ESM.docx]

**Supplemental Information: Educating engineering students to address bias and discrimination in collaborative work and decision-making**

**Chart 4: Students’ appreciation of the CAMPUS workshop (per exit survey)**

Note: Total n= 269 (of 272 campus participants), missing or no opinion responses are excluded in this chart. The general (ie non-engineering) utility question was only used in March 2020 and so has a total possible n= 61. Words in square brackets are included to facilitate the reader’s comprehension and were not part of the original items presented to students.

**Chart 5: Students’ appreciation of the ONLINE workshop (per exit survey)**

Note: Total n=44 (of 106 online participants), missing responses are excluded in this chart. Words in square brackets are included to facilitate the reader’s comprehension and were not part of the original items presented to students.

**Table 5: Online workshop participants’ utility judgements were higher than campus workshop participants (Independent groups t-test)**

| **Exit questionnaire items** | **number of respondents** | **x** | **t** | **p or t test**  **sig.** |
| --- | --- | --- | --- | --- |
| I am quite likely to try to apply the [reactive] *Make It Awkward* skills and ideas in practice. | 244 | 3.14 | -3.067 | .002* |
|  | 40 | 3.53 |  |  |
| I intend to apply some of the [proactive] decision making and discussion strategies in my current project. | 244 | 3.29 | -2.460 | .015* |
|  | 34 | 3.58 |  |  |
| The [proactive] *Space Ark* activity and the readings helped me to see where bias occurs in decisions or interactions. | 244 | 3.03 | -1.460 | .145 |
|  | 33 | 3.21 |  |  |

Note: n = 269 for campus and 44 for online questionnaires. * indicates p<.05. Words in square brackets are included to facilitate the reader’s comprehension and were not part of the original items presented to students.

**Table 6: Campus workshop participants who did return the follow-up questionnaires are representative of the larger group of campus participants (independent groups t-test)**

| **Exit questionnaire items** | **number of respondents / non- respondents** | **x respondents / x non- respondents** | **t** | **p-t-test**  **sig.** |
| --- | --- | --- | --- | --- |
| I am quite likely to try to apply the [reactive] *Make It Awkward* skills and ideas in practice. | 54 / 190 | 3.28 / 3.10 | 1.505 | .13 |
| I intend to apply some of the [proactive] decision making and discussion strategies in my current project. | 56 / 188 | 3.32 / 3.28 | .338 | .73 |
| The [proactive] *Space Ark* activity and the readings helped me to see where bias occurs in decisions or interactions. | 56 / 189 | 3.12 / 3.00 | 1.174 | .24 |

Note: Total n=269, returned questionnaires n=59, non-returned n=210. Words in square brackets are included to facilitate the reader’s comprehension and were not part of the original items presented to students.
